# Immunochemical Methods for Ochratoxin A Detection: A Review

**DOI:** 10.3390/toxins4040244

**Published:** 2012-04-13

**Authors:** Eline P. Meulenberg

**Affiliations:** ELTI Support VOF, Ambachtsweg 5, 6581 AX Malden, The Netherlands; Email: E.Meulenberg@eltisupport.nl; Tel.: +31-(0)6-1650-3626

**Keywords:** ochratoxin A (OTA), detection, quantification, immunochemical methods

## Abstract

The safety of food and feed depends to a great deal on quality control. Numerous compounds and organisms may contaminate food and feed commodities and thus pose a health risk for consumers. The compound of interest in this review is ochratoxin A (OTA), a secondary metabolite of the fungi *Aspergillus* and *Penicillium*. Due to its adverse health effects, detection and quantification are of utmost importance. Quality control of food and feed requires extraction and analysis, including TLC, HPLC, MS, and immunochemical methods. Each of these methods has its advantages and disadvantages. However, with regard to costs and rapidity, immunochemical methods have gained much interest in the last decade. In this review an introduction to immunochemistry and assay design will be given to elucidate the principles. Further, the application of the various formats to the detection and quantification of ochratoxin will be described, including the use of commercially available kits.

## 1. Introduction

Ochratoxins belong to the group of mycotoxins that are produced as secondary metabolites by fungi, in particular *Aspergillus* and *Penicillium*. These fungi flourish under special conditions of temperature and humidity. Ochratoxins include ochratoxin A (OTA), ochratoxin B (OTB), ochratoxin C (OTC) and ochratoxin α (OTα), of which OTA is considered the most toxic. They are teratogenic, mutagenic, hepatotoxic, nephrotoxic and immunesuppressive, and thus pose a serious health problem for exposed humans and animals. Because the fungi infest several kinds of crops for human and animal consumption, the metabolites may be present in all kinds of raw agricultural materials, commodities and beverages. Due to their toxic properties regulations for mycotoxins, including ochratoxins, have been established, at this moment in 100 countries and readjusted in the course of time [1,2,3,4]. Consequently, food/feed quality control is extensively performed. Analysis includes the mouse bioassay, TLC, GC, HPLC, MS and various immunochemical methods, generally after extraction of the target compound(s). Conventional analytical methods and extraction methods are beyond the scope of this review and have been described elsewhere [1,5,6,7]. In the present review the focus is on immunochemical methods for OTA analysis. However, because IAC (immunoaffinity chromatography) uses antibody for additional purification and concentration, this technique will also be described herein. Additional reviews covering conventional analytical techniques for mycotoxins, including immunochemical techniques, were published by Krska *et al.* [8] and Turner *et al.* [9]. 

The ubiquitous presence of OTA has been assessed in assay investigations and national surveys of raw and processed agricultural and derived products (cereals, food, feed, coffee, wine, beer, juices, cow milk), and the matrix has been taken into account in the development of detection methods [10,11,12,13,14,15,16,17,18,19,20,21,22,23,24,25,26,27,28,29,30]. Ingestion due to consumption has been deduced from levels found in bodily fluids and tissues in humans and animals [10,19,22,31,32,33,34,35,36,37,38,39,40,41,42,43,44,45,46,47,48,49,50,51]. Monitoring of food/feed for the presence of mycotoxins/OTA and disposal of contaminated products should lower human and animal health risk.

Immunochemical detection methods vary from simple immunoassay to highly sophisticated immunosensors. Because immunochemical methods are principally all based on antibodies, this review starts with an overview of conventional production methods of antibodies, the advantages and disadvantages as well as advanced production of antibodies and fragments thereof. Then the various formats of immunoassays will be discussed, wherein examples for the application in ochratoxin detection will be given, although not exhaustive. For further reference, see the reviews of Zheng (2006) [52] and Goryacheva *et al.* (2009) [53] for immunochemical methods for mycotoxins, including OTA. A review of available immunoassays kits was given by Huybrechts and Tangni (2010) [54]. In order to evaluate the suitability of immunochemical assays, there are several points to consider. First, the antibody/assay should meet the conditions for a reliable analytical method as with any method. For immunochemical methods, ISO norms have been established (ISO 15087). Second, the norms for the presence of the target compounds in the matrix/product to be measured should be taken into account with regard to the detection limit and working range of an assay. Validation of a newly developed immunoassay also requires reference materials, which may be difficult to obtain, especially in the case of highly toxic and/or complex compounds. For OTA in agricultural products such reference materials are available now. In this review the development, design, evaluation and use of immunochemical methods for the detection and/or quantification of OTA are described. Special attention will be given to chemical/synthetic “antibodies”.

## 2. Antibodies

**Figure 1:**
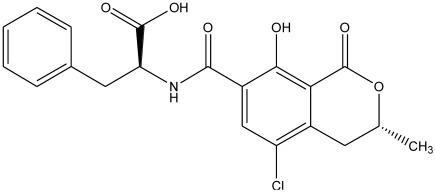
Chemical structure of ochratoxin A.

**Figure 2:**
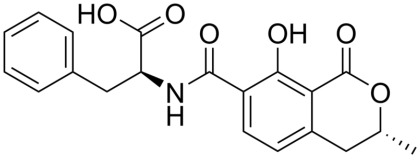
Chemical structure of ochratoxin B.

The antibody forms the core component of any immunochemical method, because it is the element that recognizes and binds its target compound (antigen). Antibodies are components of the immune system of animals that defend the body against intruding substances and organisms. They are produced by specialized cells of the immune systems and they comprise several forms: IgA, IgD, IgE, IgG, IgM, IgY (avian). The predominant form secreted in blood is IgG and this form is generally used in immunochemistry. The production of antibodies starts with the immunization of experimental animals, such as rat, rabbit, mouse, sheep, horse, goat, chicken. To be able to raise an immune reaction, the injected compound (immunogen) has to meet several conditions: >1000 Dalton, foreign for the body and with a 3-dimensional structure. In the case of a small compound (hapten), such as ochratoxin, the particular compound is generally coupled to an immunogenic protein, optionally via a spacer group. Coupling proteins include bovine serum albumin (BSA), keyhole limpet hemocyanin (KLH), thyroglobulin (TG), polylysine, among others, although BSA is predominantly used. Coupling procedures are known from literature. Generally, when a hapten belongs to a group of related compounds, the coupling to a carrier protein is performed such that the moiety unique for that hapten is exposed and the carrier protein is bound to another site of the compound. In the case of OTA, the free carboxylic group is commonly used for coupling because of easy chemistry (Figure 1). As will be clear from the section below, this provides antibodies with low cross-reactivity to related compounds. Reversibly, the most resembling mycotoxins OTB (Figure 2) showing sometimes cross-reactivity in OTA immunoassays, provides, when coupled in the same way, antibodies highly specific for OTB [55].

Immunization involves primary injection of the immunogen, followed by several booster injections. After about 2–6 months the titer (concentration) of the desired antibody is, in general, sufficiently high for use in an assay. The serum of the animal may be used as such, but in most cases the antibodies are isolated and purified with standard methods. Immune serum or purified antibodies are designated polyclonal antibodies (PAb), because they comprise a population of antibodies with different affinities and specificities [56].

Another widely used production method involves the hybridoma technique [56,57]. Traditionally, the immunogen is used to immunize mice, although nowadays other species are also used. After 2–3 boosters, the spleen is isolated, processed to splenocytes which are fused with myeloma cells, and cultured in limited dilution so that each single antibody producing cell will give rise to a separate cell culture (hybridomas). Then the best performing culture is chosen for mass production and isolation of the antibody. Such antibodies are designated monoclonal antibodies (MAb). In contrast to polyclonal antibodies, monoclonal antibodies consist of one type of antibody with defined affinity and specificity. In addition, monoclonal antibodies can be produced as long as a hybridoma is viable. Sometimes monoclonal antibodies are less stable than polyclonal antibodies.

Alternative forms of antibodies include recombinant antibodies, phage displayed antibodies, plant antibodies, and antibodies fragments that have retained their antigen binding domain. For production methods and properties, there have been published numerous articles and reviews [58,59,60,61,62]. However, these have until now not been used for the development of immunoassays for OTA.

Once an antibody has been obtained, it has to be characterized with regard to specificity and affinity for use in an assay to be suitable for the detection of its target compound. Importantly, in order to have high specificity in an assay, the properties of the antibody with regard to cross-reactivity to related compounds should be determined in subsequent assays. Closely related compounds comprise OTB, OTC, OTD, OTα, coumarin, OH-coumarin. Because of the small size of these compounds, they fit into the binding pocket of the antibody and their common moieties (epitope, binding part) may lead to more or less affinity for the antibody and thus cross-reactivity. Due to a completely different structure, other mycotoxins that are often found on and/or in crops as co-toxins generally will show no cross-reactivity and thus no interference in an immunoassay.

## 3. Immunoassay Formats

The immunochemical reaction, *i.e.*, the binding of antibody and antigen, in an assay is not visible and therefore several means to detect the reaction product, the immune complex, have been developed based on signal-generating components and appropriate measuring devices. The various immunoassays are named based on the signal-generating component or tracer.

### 3.1. RIA (Radioimmunoassay)

The first immunoassays made use of a radioactive tracer consisting of the target compound incorporated with a radionuclide, such as ^3^H, ^14^C, ^125^I, although other radionuclides may also be used. The principle of a RIA is the competition between labeled and unlabeled compound for a limited number of binding sites on the antibody. The more unlabeled target compound in a sample, the less tracer is bound to the antibody and radioactivity counting yields a measure for the concentration of target compound in a sample, based on a series of standards. It will be appreciated that ^3^H and ^14^C will hardly influence the physic-chemical properties of the tracer and its behavior will hardly change, in contrast to ^125^I, which is a larger moiety. In order to be able to count only the radioactivity of the immune complex, a separation step is needed. For small compounds, dextran coated charcoal has often been used to perform this separation, followed by centrifugation. Larger target compounds in complex with their binding antibody may be separated using e.g., PEG, followed by centrifugation. An alternative separation method is the use of equilibrium dialysis (ED) where only the free fraction is passing a membrane.

In earlier days, ochratoxin was analyzed by RIA using polyclonal antibodies in several formats. For example, Aalund *et al.* (1975) [63] used OTA-bovine IgG as an immunogen to produce polyclonal antibodies and ^125^I-ovalbumin-OTA as a tracer, followed by a precipitation step. The detection limit was 20 µg/L, which is quite high. This may be due to the large difference between the tracer that includes ovalbumine, a relatively large protein, and OTA itself. The use of ^3^H-OTA as a tracer in combination with ED for separation of bound and free fraction, was described by Chu *et al.* (1976) [64]. Here the detection range was from 1–20 ng/mL. Rousseau *et al.* (1985) [65] developed a ^14^C-based RIA in combination with a polyclonal antibody for the detection of OTA in barley. The detection limit was 0.5 ng/mL and the antibody was rather specific for OTA, showing low cross-reactivity with related compounds. In a further study this group also analyzed OTA in serum with a detection limit of 0.4 ng/mL [66]. A ^125^I-based RIA kit was used by Fukal and Reisnerova (1990) [10] to assess the ingestion of OTA by humans by measuring OTA in serum (detection limit 100 ng/L). Their conclusion was that OTA is actually ingested and present in serum. Fukal (1990) [67] reported that, based on measurements using the same kit, food and feed may contain OTA, but it was not detected in porcine kidney.

Due to health hazards of radiolabeled compounds and specialized waste disposal, RIA has not been in use for a long time. Advanced immunochemical methods included various alternative tracers and recently even label-free methods.

### 3.2. EIA (Enzyme Immunoassay)/ELISA (Enzyme-Linked Immuno Sorbent Assay)

A great advancement in immunoassay was the replacement of radionuclides by enzymes in combination with a substrate that is converted into a detectable colored product. There are many examples of possible enzymes, but HRP (Horse Radish Peroxide) and ALP (Alkaline Phosphatase) are used predominantly. The development of EIA was accompanied by the application of solid supports such as plastic reaction tubes or microtiter plates whereon immunoreagents are coated and the immunochemical reaction is performed (ELISA). After the reaction, the supernatant solution containing unbound fraction is discarded and the signaling enzyme reaction is performed. In such formats the steps of adding separation means and centrifugation are avoided, which shortens the assay time.

ELISA may be designed in various formats. The simplest formats are the direct competitive ELISA (dcELISA) and indirect competitive ELISA (icELISA). The former involves the coating of antibody onto the walls of a tube or microtiter plate, adding standard/sample, followed by antibody and tracer. After incubation, the reaction solution is discarded and the enzyme reaction is performed, which is analyzed with spectrophotometry. In the icELISA the hapten, generally coupled to a carrier protein, is coated onto the support, standard/sample is added, followed by labeled antibody. Further reaction steps are similar to the dcELISA. Between the reaction steps extensive washing is included.

There exist various variants of this kind of enzyme immunoassay, such as the icELISA wherein a secondary antibody raised against the primary antibody, for example, anti-rabbit IgG for polyclonals or anti-mouse IgG for monoclonals, is labeled. Secondary antibody may also be used as a first coating layer to capture primary antibody, where the reaction takes place. Further alternative formats are described in literature and the reader is referred to a recent review [68].

ELISA has been the preferred method to detect and quantify OTA in raw products, food, feed, beverages, bodily fluids and tissues to assess its presence and concentration for either exposure risks, quality control and as a measure of ingestion and accumulation. Table 1 provides some data found in literature. Formats used for OTA other than ELISA are discussed in further sections below.

**Table 1:** ELISA for OTA.

Year	Antibody	Format	Matrix	D.L.	Validation	Reference
1981	PAb ^1^	dc	buffer	25 pg	cr	[69]
1988	MAb ^1^	ic	barley	5 µg/kg	−	[70]
1989	MAb ^1^	ic	various	50 pg/mL	+	[71]
1990	MAb ^1^	dc	barley	1 ng/mL	+	[72]
1991	MAb ^1^	dc	barley	1 ng/mL	+	[73]
1996	MAb ^1^	dc	cereals	0.5 ng/g	+	[74]
1996	MAb ^1^	dc	buffer	45 pg/mL	cr	[75]
1997	MAb ^1^	dc	serum	0.2 ng/mL	+	[34]
1998	MAb ^1^	dc	plasma	4–20 pg/mL	−	[38]
1999	MAb ^2^	m	wheat	0.4 ng/mL	+/−	[76]
2000	MAb ^1^	ic	chilies	5 ng/mL^a^	+	[77]
2002	MAb ^1^	dc	plasma/tissue	0.5 ng/g	+	[43]
2002	MAb ^2^	m	coffee	4 µg/kg	cr	[78]
2006	MAb ^1^	ic	coffee	50 pg/mL	+	[28]
2007	MAb ^1^	ic	coffee	3.73 ng/g	+	[79]
2007	PAb ^1^	m/dc	chilies	10 µg/kg	+	[80]
2007	PAb ^1^	dc	various	1 ng/mL	+/−	[81]
2009	MAb	str	corn	2.5 ng/mL	−	[82]
2011	PAb ^1^	str	grains	1.5 µg/kg	+	[83]
2011	MAb ^1^	ic	cereals	1.70 ng/mL ^a^	−	[84]

^1^ = produced in-house; ^2^ = commercial; m = membrane; ic = indirect competitive; dc = direct competitive; str = strip test; ^a^ = IC_50_; cr = only cross-reactivity; + = validated; − = not validated; +/− = partially validated.

In-house production of antibodies is a tedious procedure and requires facilities that are not available to each lab. For the development of an immunoassay commercially available PAb or MAb may be used. Although ELISA is basically a simple analytical method that does not require highly trained technicians, the development, design and validation does. Therefore, as an alternative, immunochemical analysis may be performed with commercial ELISA kits. Use of an ELISA kit for the analysis of wheat flour (Turkey) revealed that 83% of the samples contained OTA [25]. An OTA ELISA kit in combination with an IAC kit was validated for analysis of OTA in wine. The detection limit was 0.054 µg/L, working range 0.25–9 µg/L; the recovery 94–102%; and results compared good with HPLC. A survey of the presence of mycotoxins in herbs (Spain) using commercial IAC columns and ELISA kits with a detection limit for OTA of 0.025 µg/kg showed that 99% of the samples were contaminated [30].

Although somewhat less toxic, OTB is quite similar to OTA and is often a co-contaminant. To be able to detect OTB in the same samples, a MAb and an ELISA were developed by Heussner *et al.* (2007) [54]. The same strategy as for OTA was used, *i.e.*, coupling of carrier proteins to the carboxylic moiety. A detection limit of 27 nM was reported, with low cross-reactivity (3.3%) for OTA.

### 3.3. Sandwich ELISA

For larger target compounds containing several epitopes, the sandwich ELISA is a convenient format. Herein the solid support is coated with one antibody raised to one epitope of the compound; target compound is added, followed by a second antibody raised against another epitope of the compound. Second antibody may be labeled or a third labeled antibody may be used for detection. Because OTA has a low molecular mass and fits into the binding pocket of antibody, there is no further moiety exposed for binding of second antibody. However, a sandwich ELISA system has been developed to detect several molds (*Aspergillus*, *Penicillium*, *Fusarium*) simultaneously by using monoclonal antibodies against the extracellular polysaccharide of these species [85]. Interestingly, Punyatong *et al.* (2003) [86] described a sandwich ELISA using two monoclonal antibodies produced in-house with OTA-HSA as immunogen. The developed ELISA, one MAb coated on microtiter plate and one HRP-labeled MAb, showed 50% binding at 35 pg/assay, which is quite sensitive. No further validation or application has been described.

### 3.4. Chemiluminenscent Immunoassay (CL-IA)

Some samples show high matrix effects in EIA and dilution may be a means to eliminate them. However, that means that the assay should be more sensitive. According to Yu *et al.* (2011) [87] a CL-IA may offer an elegant solution. They designed an assay using soy bean peroxidase in combination with luminol and an enhancer (3-(10'-phenothiazinyl)propane-1-sulfonate/4-morpholinopyridine) and they achieved a limit of detection (LOD) of 0.01 ng/mL and a working range of 0.02–0.3 ng/mL in several agricultural products. When samples were compared to a direct competitive ELISA, it appeared that in some samples being negative in the ELISA, OTA could be detected. These CL-IA results were comparable to those of Qui (2010) [88]. Herein an indirect CLIA with HRP-labeled secondary antibody provided a simple, fast and sensitive screening assay for corn samples with an LOD of 0.04 µg/kg.

### 3.5. Fluorescent Immunoassay (FIA)

Fluorescent immunoassays are a variant of immunoassays, wherein the tracer contains a fluorofore as a label. The advantage of fluorofores is their broad range of excitation and emission wave lengths and availability. However, FIA requires special equipment and microtiter plates (black or white), and care should be taken to avoid background fluorescence interference. The design of FIAs may be direct (labeled hapten), indirect (labeled primary antibody and with labeled secondary antibody. Although simple FIA has not been used for analytical purposes, there exist some variant FIA, which are described below.

#### 3.5.1. Time-Resolved Fluorescent Immunoassay (TR-FIA)

The characteristic of TR-FIA is the use of fluorofores with a longer fluorescence life time, which eliminates background fluorescence and thus enables a more sensitive and specific assay [89]. This technique forms the core in Delphia immunoassays [90], but many other formats and applications are possible. The use of lanthanide labels in TR-FIA with luminescence detection has been reviewed by Hagan and Zuchner (2011) [91]. In addition, when using two different labels, multi-analyte immunoassay is feasible. An example of mycotoxins detection has been given by Huang *et al.* (2009) [92] who developed a TR-FIA for OTA and Aflatoxin B1 (AFL B1) using Sm and Eu as a label, respectively. In this format, antigen-protein was coated onto microtiter plates, then sample and antibody (MAb for OTA and PAb for AFL B1) were added, followed by differently labeled second antibody. This TR-FIA was validated and showed a detection limit for OTA of 0.05 µg/L (range 0.05–50 µg/L) and for Aflatoxin B of 0.02 µg/L (range 0.02–100 µg/L).

#### 3.5.2. Fluorescence-Polarization Immunoassay (FP-IA)

Another variant fluorescent immunoassay is FP-IA. The principle of this technique has been given in a review of Maragos (2009) [93], including a table showing results from literature for mycotoxins. In short, the binding of a hapten to its antibody is detected by measuring the change in tumbling motion of the particular fluorofore resulting in a change in observed polarization. The solution containing the immunoreagents is exposed to plane-polarized light and the emission light is separated by a vertical and horizontal polarizer. The advantage of FP-IA is that the complete assay is performed in solution, avoiding washing and separation of bound and free fraction. An example of the application of FP-FIA can be found in a publication of Zezza *et al.* (2009) [94]. This group measured OTA in red wine using both their own and a commercial MAb. The assay was validated with spiked samples and compared to IAC/HPLC. A detection limit of 0.7 ng/mL (below the EU norm of 2.0 ng/mL) was achieved. 

#### 3.5.3. FRET (Fluorescence Resonance Energy Transfer) Immunoassay

The technique of separation-free, homogeneous FRET immunoassay is described by Kreissig *et al.* (2011) [95]. Such an assay uses electronic excited state energy transfer. Both the tracer and the antibody are labeled with a fluorescent tag, a donor and an acceptor respectively. When binding occurs fluorescence is quenched, but displacement by the target compound eliminates this quenching and fluorescence can be measured. A FRET assay, wherein the binding of OTA to its antibody is detected, was developed by Li *et al.* (2011) [96]. After optimizing reaction conditions, an LOD of 1 ng/mL was achieved, comparable to other immunoassay formats and a commercial kit. There was no cross-reactivity with OTB and the recovery in spiked wheat samples was around 100%.

#### 3.5.4. Fluorescent Micro-Array Immunoassay

An alternative immunoassay format for OTA has been described by Ngundi *et al.* (2005) [97]. Here a pretreated glass slide is functionalized with NeutrAvidin. OTA-biotine conjugate is patterned onto the slide and then sample plus labeled (Cy5, fluorescent) antibody (PAb, commercial) are reacted by addition through flow channels. After the immunochemical reaction the slide is analyzed by fluorescence CCD imaging. The detection limit varied from 3.8–100 ng/g for various samples of cereals, macaroni, coffee and wine. A feasibility study into the development of a multi-analyte rapid screening microarray device was described by Lamberti (2009) [98]. Herein the presence of both aflatoxin B1 and fumonisin B1 could be detected on BSA-conjugates bound to functionalized (co-polymer) glass slides. This design holds promise for future adaptation to include other mycotoxins, including OTA.

### 3.6. Lateral Flow Immunoassay (LF-IA)

Rapid screening of samples for contaminants, including OTA, may be performed using lateral flow immunoassay, a form of paper immunochromatography and also called strip tests. A review of the technique was recently reported by Posthuma (2012) [99] and the application was reviewed by Bazin *et al.* (2010) [4]. 

LF-IA relies on the movement of immunoreagents over a porous material containing pads or lines of target conjugate, sample and antibody, wherein either the target conjugate is labeled or the antibody. The principle of LF-IA is the same as in an EIA. The label may be a chromofore, but nowadays labels consisting of nanoparticles are preferred. The most simple format is the dipstick (mentioned in the reviews of Prieto-Simon and Campas (2009) [29] and Goryacheva *et al.* (2009) [53]). In fact, with haptens as target compounds only the indirect format is feasible. Thus to the porous material are added a hapten-conjugate pad and optionally a test line with secondary antibody, preceded by a release pad and followed by an absorbent pad. Sample and labeled antibody are mixed and added to the release pad and then passed through the porous material by capillary force, competition reaction takes place at the conjugate pad where the signal evaluated visually or measured with appropriate devices. 

LF-IA may be designed for multi-analyte detection. For example, using Au-nanoparticles coupled to MAb’s was applied and validated by Shim *et al.* (2009) [82] for the detection of both OTA and zearelanone in spiked corn samples. The visual detection limits were 2.5 and 5 ng/mL, respectively. Results were compared to dcELISA and HPLC. A similar LF-IA was developed by Anfossi *et al.* (2011) [83] using commercially available PAb that were coupled to Au-nanoparticles. Here nitrocellulose strips were used for application of OTA-BSA lines. Detection of the immunecomplex was performed with a scanner and a detection limit of 0.15 µg/kg of cereal extracts was demonstrated. Results compared very well with LC-FLD.

### 3.7. Flow-Through Immunoassay

Immunoassays performed in microtiter plates require washing steps and discarding supernatant. To simplify this format, membrane-based immunoassays were developed. Here the antibody is coupled to a membrane attached to a well and the immunochemical reaction using enzyme-labeled antigen/hapten takes place on this membrane. Addition of substrate/chromofore leads to a visible spot on the membrane that may be evaluated visually. It is a rapid, semi-quantitative screening method and kits for OTA are commercially available. Flow-through immunoassay for OTA has been applied by De Saeger and van Pethegem (1999) [76] using MAb and OTA-HRP. The assay required 15 min of assay time and the detection limit was 0.4 ng/mL. The same working group has validated membrane-based immunoassay kits for OTA and T-2 toxin in a ring test with cereal samples (detection limit for OTA 4 µg/kg, for T-2 toxin 50 µg/kg), wherein the results were compared to HPLC and GC. Comparable results were obtained for OTA in spiked roasted coffee samples by Sibanda *et al.* (2002) [78]. Their membrane-based assay showed a detection limit of 4 µg/kg and results were confirmed with HPLC in the scope of a patent application.

A recent development of flow-through immunoassay is multi-analyte flow cytometry using the MultaAnalyte Profiling (xMAP) technique in combination with a Luminex apparatus (Austin, TX, USA). Discrimination of analytes is based on color-coded superparamagnetic beads and detection is performed with fluorescent label. The immunochemical reaction occurs in 96-wells microtiter plates, wherein washing and separation steps are performed by application of a magnet. Both a direct and an indirect format were described for the simultaneous detection of OTA and other mycotoxins. In the indirect format the beads a coupled with OTA-conjugate, reacted with sample and antibody or biotinylated antibody. For signal generation, secondary antibody-PE (phycoerythrin) or streptavidin-PE is used, respectively (Peters *et al.* 2011, Anderson *et al.* 2010) [100,101]. The detection limits found were in the range of 10–30 ng/g for spiked cereals. A direct format, wherein antibody is coupled to the beads and the reaction is performed by adding sample and OTA-PE conjugate for competition assay is described by Aqai *et al.* (2011) [102] and showed a detection limit of 0.15 ng/g. The sensitivity of the Luminex method and multi-analyte mode makes it superior to other immunochemical methods. 

## 4. Immunosensors

Sensors comprise devices for real-time, on-site detection of target compounds. Biosensors use a biomolecule as core component and in immunosensors an antibody is the recognition element, wherein the binding to its cognate ligand leads to a detectable signal. Immunosensors exist of a reaction surface, a transducer and a detector. Transducers may be electrochemical, optical and gravimetric (acoustic wave (AW)/quartz crystal microbalance (QCM)). The techniques have been explained in several reviews [103,104,105,106,107,108]. The most commonly used immunosensors are based on optical transducers and surface plasmon resonance (SPR) is the most known and utilized. Devices generally comprise the Biacore (Uppsala, Sweden) which are available in several embodiments. Even a portable SPR system, the Spreeta, has been mentioned for rapid field mycotoxins analysis [109]. Toxin detection with SPR was reviewed by Hodnik and Anderluh (2009) [110]. Practical application of SPR sensing for OTA was described by Adányi *et al.* (2007) [111] for the parallel determination of aflatoxin B1 and OTA in barley and wheat flour samples. The immunochemical format was competition immunoassay with hapten-BSA immobilized on the sensor surface and the use of specific monoclonal antibodies in combination with sample flowing over the surface for measurement. This format revealed a detection range between 0.5 and 10 ng/mL. A similar SPR assay for OTA, but with highly improved sensitivity was reported by Yuan *et al.* (2009) [112]. They used a new conjugate, OTA-PEG-BSA, for immobilization and Au-nanoparticle coupled monoclonal antibody to achieve much better performance, enabling a range of LODs from 0.058–0.4 ng/mL in cereals and beverages. Second most used immunosensors are based on electrochemical detection. Various parameters for the design of an electrochemical immunosensors for OTA in an indirect format were investigated by Prietó-Simon *et al.* [113], such as the coating conjugate (OTA-BSA/avidin-OTA), polyclonal or monoclonal antibody, and enzyme-conjutate (OTA-HRP/OTA-ALP). In the best performing and most stable embodiment a detection limit of 0.7 ng/mL was achieved. In a comparable format using OTA-BSA, monoclonal anti-OTA, but ALP-labeled secondary antibody for detection a very low detection level of 8.2 pg/mL was found [114]. This sensor performed also well in corn samples for matrix effects. A direct format in an electrochemical immunosensors using polyclonal antibody on a modified gold electrode in conjunction with OTA-HRP provided a detection limit of 12 ng/mL [115]. Recently, an electrochemical competitive immunosensor with a detection limit of 0.10 ng/mL for OTA, validated using certified wheat samples, was described by Vidal *et al.* (2011) [116]. Performance was improved by using OTA-BSA on Au-nanoparticles on the sensor surface, biotinylated monoclonal antibody as binding component and extravidin-HRP for signal generation. An even lower detection limit, 60 pg/mL, was achieved by Urusov *et al.* (2011) [117] by signal enhancement due to use of second antibody-colloidal gold particle conjugate. The application of magnetic nanoparticles (MNP), which show various advantages in terms of surface area, stability of bound antibodies, improved orientation of the antibodies and fast assay kinetics, was described by Zamfir *et al.* [118]. The design comprised a gold surface containing several layers of self-assembling monomers, BSA and MNPs coated with MAb. Detection was performed with both electrochemical impedance spectroscopy (EIS) and SPR, providing a detection limit of 0.01 ng/mL and 0.94 ng/mL, respectively. Results of the assay of spiked white wine were compared to ELISA.

## 5. Synthetic/Chemical Antibodies

A rather new development in binding chemistry and assays comprises synthetic or chemical “antibodies”, such as MIPs (molecularly imprinted polymers) and aptamers (single-stranded oligonucleotides). MIPs are synthetic polymers with selectivity and specificity for a particular target compound. They are synthesized by mixing target compound and (vinyl) monomers in suspension and then perform polymerization, followed by removal of the target compound. What remains are structures that can specifically harbor the target compound and may be used as alternatives to antibodies in the same applications. The synthesis of MIPs is illustrated in a review of Whitcombe and Vulfson (2001) [119]. A comparison between MIPs and antibodies is given by Lavignac *et al.* (2004) [120], indicating affinities of MIPs being lower than of antibodies, but having the advantage of assays to be performed in non-aqueous conditions. They also describe the characterization of radio-molecularly, fluoro-molecularly enzyme-linked molecularly imprinted sorbent assays for a range of small compounds, including non-immunogenic analytes. The application of MIP-based solid phase extraction (MIP-SPE) for environmental pollutants from water, soil and tissues, as well as MIP-based sensors combined with various transduction methods for environmental analytes, including the mycotoxins zearalenone, has been reviewed by Pichon *et al.* (2008) [121]. Although this technique has not been described for OTA, it is a promising development both for extraction and detection purposes.

Aptamers are another form of chemical binding entities. They are short single-stranded DNA or RNA ligands containing 10–50 variable bases. Aptamers and opportunities for application were discovered already more than 20 years ago. Since that time several reviews have been published about principles and production [122], signaling in aptamer-based biosensors, also called aptasensors [123], electrochemical (EC) aptasensors [124], analytical/pharmaceutical applications [125,126,127], homogeneous assays with aptazymes wherein aptamers regulate the activity of DNA/RNAzymes [128] and aptasensor with fluorescence detection [129,130]. In practice, having synthesized a DNA or RNA library, aptamers against a particular target are selected by the procedure called SELEX (Systematic Evolution of Ligands by Exponential enrichment). Herein the library is incubated with target, the DNA/RNA-target complex is isolated and eluted and the eluted DNA/RNA is concentrated and amplified. Once a desired aptamer has been obtained, it may be used in formats similar to immunoaffinity columns, immunoassays and immunosensors using comparable sensing techniques. With regard to OTA, an aptamer specific for OTA was applied to an affinity column to capture OTA from wheat sample extracts with known concentration, followed by fluorometric analysis [131]. An ELISA-like enzyme-linked aptamer assay (ELAA) in the direct and indirect format for the detection of OTA was developed by Barthelmebs *et al.* (2011) [132] and evaluated for analysis of spiked red wine samples. The method, especially the direct format, compared well to MAb-based direct/indirect ELISA and showed a detection limit of 1 ng/mL with an analysis time of 125 min, which makes it a useful screening method for routine use. Aptasensors for mycotoxins with electrochemical detection were developed by several groups. For example, Kuang *et al.* (2010) [133] designed and OTA aptasensor including 3 DNAs, DNA1 for capturing DNA2 (OTA aptamer) on the sensor surface and DNA3 on gold nanoparticles (AuNP) for EC detection using methylene blue for detection of OTA binding to the aptamer and signal amplification. Aptamer coupled to AuNP was used by Bonel *et al.* (2011) [134] for EC detection in a competitive assay with OTA-HRP. The sensor was validated with spiked wheat samples and showed a detection limit of 0.07 ng/mL with a range of 0.78–8.74 ng/mL. A different EC aptasensor was described by Tong *et al.* (2011) [135]. Herein a DNA partly complementary to the OTA aptamer was bound to AuNP for hybridization of the aptamer. Binding of OTA dissociates the aptamer-OTA complex. They achieved a detection limit of 1.0 pg/mL and a range of 0.005–10.0 ng/mL. This aptasensor was validated with spiked and real wheat starch samples in comparison with an ELISA. An aptasensor based on conformational change of aptamer induced by binding of OTA leading to aggregation of the AuNP used for aptamer coupling was described by Yang *et al.* (2011) [136]. Here a detection limit of 2.5 nM with a range of 20–625 nM was obtained. DNAzyme was applied in the aptasensor developed by Yang *et al.* (2012) [137]. Binding of OTA to aptamer induced hairpin opening resulting in enzyme activity of HRP-mimicking DNAzyme and oxidation of TMB, which could by measured colorimetrically. Signalling using quantum dots (QD) was applied in a fluorescent strip sensor, wherein QD were conjugated to OTA aptamer and the conjugates applied to strips similar to LF-IA. After the reaction the reading is visually with a limit of detection of 5 ng/mL within 10 min, and 1.9 ng/mL when using Image Analysis Software for a calibration curve in spiked red wine samples. The results were compared to HPLC for confirmation.

## 6. Immunoaffinity Chromatography

A long used application of antibodies is in immunochromatography for the capture, isolation and/or concentration of target compounds. In this case an antibody is covalently coupled to a solid support (e.g., functionalized agarose, sepharose, silica) and transferred into a column. When sample containing target compound is passed through the column, the target compound will bind to the antibody and may be eluted after washings that remove unwanted matrix components. An elution volume smaller than the sample volume, allows for concentration of the target compound. Commercial IAC columns are available for many target compounds, including OTA, and used as a purification means before further analysis (HPLC, MS, ELISA). A combination of IAC and immunoassay for OTA in one column was developed by the group of van Peteghem (Ghent University) in cooperation with the Saratov State University (Moscow). Initially, the column consisted of a layer of antibody-coupled solid support upon a clean-up layer. OTA-HRP was used for the competitive immunochemical reaction that was detected with TMB and visually evaluated. In samples of roasted coffee [138] and spices [139] a cut-off value of 6 µg/kg and 10 µg/mL, respectively, was demonstrated. By using two different antibody containing layers in a similar IAC column both OTA and aflatoxin B1 could be detected in spices [140]. As for the analysis of red wine the cut-off value according to EU legislation is lower than for coffee or spices, the method was optimized to a lower sensitivity (2 µg/L). In this embodiment a separate clean-up column was placed upon a flow-through column containing antibody [141], and further designed for the simultaneous detection of 2,4,6-trichlorophenol and OTA in red wine or spices [142,143] at a level of 2 µg/L and 10 µg/kg, respectively.

Immunoaffinity extraction in a different setting has been described by Aqai (2011) [102]. Herein monoclonal anti-OTA antibody was coated onto magnetic beads for the capture as well as identification of the target compound in competition with OTA-PE. Detection in wheat and cereal sample extracts was performed by flow cytometry (see above) and compared to LC-MS.

Chemical antibodies have also been used for OTA extraction and analysis. However, due to low recoveries onto MIP, specific extraction had to be preceded by SPE extraction to remove interfering substances. Analysis included LC-MS/MS. Aptamer as selective capturing moiety in an affinity column for the determination of OTA in wheat grain has been described [132]. Analysis of eluted samples was performed with fluorescence spectrometry. Due to the lower affinity in comparison to antibodies, the sensitivity of this method was in the ppb range. 

## 7. Summary

When monitoring for the presence of OTA either in raw and derived agricultural products, there is a large choice of methods. Depending on the purpose, either rapid detection or validation according to the regulations, one can use quantitative and qualitative methods. Among the available conventional methods, HPLC, and among the immunochemical methods, ELISA have traditionally been applied. If performed according to the ISO norms, ELISA may be considered as equipotential to the conventional methods, with comparable sensitivity and the advantage the several samples may be analyzed in the same run. Many researchers have developed and designed in-house antibodies and ELISA. However, commercially available kits have the advantage of avoiding long development which can take a few months. In the course of time there have been successful attempts to improve the ELISA with regard to sensitivity and analysis time. First, the tracer was changed from enzyme plus chromofore to fluorofore. The advantages of using fluorofores are: an improved sensitivity may be achieved; by applying different fluorofores a multi-analyte assay may be designed (TR-FIA); and even homogeneous immunoassays are possible, avoiding washing and separation steps (FP-IA, FRET (label-free)). Disadvantages of fluorescent assays are that background fluorescence may interfere and should be eliminated, and the required equipment is more expensive. Chemiluminescent immunoassay is a variant of ELISA wherein a comparable enzyme is combined with luminol and an enhancer. CL-IA is a rather new technique for OTA detection, but due to a high sensitivity (0.01 ng/mL) and applicability for screening purposes, CL-IA looks promising. Advanced label-free detection methods include biosensors such as SPR (optical) or gravimetric immunosensors, wherein the binding of antigen to antibody is detected by a change in plasmon resonance or mass. SPR may be performed in direct and indirect format and can easily be used for multi-analyte analysis. Improvements include the use of Au-nanoparticles enabling detection limits down to 0.06 ng/mL. Despite the usefulness, SPR equipment is quite expensive. Depending on the number of steps and costs, electrochemical immunosensors may be a good alternative for rapid detection of OTA in various matrices.

Qualitative detection methods include membrane-based immunoassays, lateral flow systems and IAC columns. Each of these is commercially available and may be used for a rapid screening of samples. Reading is visual, although the results may be quantitative when using appropriate devices, such as a scanner. Improvements in strip tests were achieved when using AU-nanoparticles and multi-analyte detection, for example, OTA and zearelanone have been described. Immunoassay on IAC columns is a useful variant of IAC that may be used in the field for both single- and multi-analyte detection. Mycotoxins, including OTA, may be detected at the cut-off level according to EU legislation in different matrices. The advantage of these quantitative methods is the ease and rapidity of performance.

A recent development in analysis includes chemical/synthetic antibodies, MIPs and aptamers. MIPs are polymers forming a kind of binding pocket for a particular target compound. At first, MIPs have proven useful to extraction purposes in SPE-like columns. An MIP for zearelanone applied in a sensor has been described in a review about MIPS for environmental application and production of an MIP-based sensor for OTA is probably only a question of time. Aptamers are oligonucleotides resembling antibodies in selectivity and affinity. They are selected from libraries and used for extraction of OTA, enzyme-linked aptamer assays (ELAA), strip tests, and aptasensors. Improvement of the ELAA by using Au-nanoparticles gave a detection limit of 0.07 ng/mL. Aptasensors show an even lower detection limit of 1.0 pg/mL in wheat samples in an embodiment using partly complimentary DNA. A rapid strip test using quantum dots for labeling of OTA and fluorescent detection showed a detection limit of 5 ng/mL in visual reading and 1.9 ng/mL with a scanner. The assay time in this case was very short (10 min). Aptamers have the advantage that the oligonucleotides, once selected and validated, may be synthesized in large amounts as required.

In conclusion, there is a wide range of immunochemical methods for OTA detection which are widely available or easy to design. Depending on the user’s detection purposes and resources, the most appropriate method may be chosen.
